# High-Pressure Processing and Ultrasonication of Minimally Processed Potatoes: Effect on the Colour, Microbial Counts, and Bioactive Compounds

**DOI:** 10.3390/molecules26092614

**Published:** 2021-04-29

**Authors:** Konstantina Tsikrika, Des Walsh, Ashik Joseph, Catherine M. Burgess, Dilip K. Rai

**Affiliations:** 1 Department of Food BioSciences, Teagasc Food Research Centre Ashtown, D15 KN3K Dublin5, Ireland; kontsikrika@gmail.com (K.T.); ashikjoseph212@gmail.com (A.J.); 2 Food Safety Department, Teagasc Food Research Centre Ashtown, D15 DY05 Dublin5, Ireland; des.walsh@teagasc.ie (D.W.); kaye.burgess@teagasc.ie (C.M.B.)

**Keywords:** nonthermal technologies, high-pressure processing, ultrasound, shelf-life, polyphenols, potatoes

## Abstract

HPP at 600 MPa alone, and in combination with US at 20 kHz (200 W), was applied to minimally processed potatoes of two commonly grown cultivars in Ireland. Changes in colour and microbial load (*Enterobacteriaceae*, total aerobic count, *Salmonella*, yeasts, and moulds) were monitored in vacuum-packaged potatoes during 14 days of storage at 4 °C. HPP and HPP/US significantly (*p* < 0.05) affected the colour parameters a*, b*, L*, and ΔE of minimally processed potatoes compared to the controls. Microbial growth was delayed in most of the treated samples with respect to those untreated (controls), while HPP completely inactivated *Enterobacteriaceae* in both cultivars. Total phenolic content and antioxidant activities were not altered in the treated samples of both varieties when compared to the controls. The levels of chlorogenic acid, ferulic acid, and caffeic acid were decreased after both treatments, with a significant (*p* < 0.05) increase in quinic acid in the treated samples as opposed to those untreated. A significant (*p* < 0.05) decrease in the levels of glycoalkaloids, namely α-chaconine and α-solanine, in HPP- and HPP/US-treated potatoes was also observed. These findings suggest that HPP and US can extend the shelf-life of minimally processed potatoes with a negligible impact on their antioxidant activity and phenolic content.

## 1. Introduction

Several methods have been employed to prolong the shelf-life of fresh-cut products while retaining their quality. Minimally processed potatoes are prone to browning reactions resulting in undesirable changes in their wholesomeness, nutritive, and organoleptic attributes. Blanching is the most common method of processing fresh-cut potatoes to prevent enzymatic browning, while at the same time promoting a more uniform colour after frying, minimizing oil absorption, and enhancing texture [1]. However, thermal treatments, such as blanching, may have an adverse impact on the nutrients and flavour, as well as on the colour of the potatoes.

Potatoes constitute a staple nutritional diet worldwide, while they are also known to contain phytochemicals that have a beneficial role in human healthincluding anthocyanins, phenolic acids, and other poly(phenolic) compounds [2,3]. The loss of these bioactive compounds, along with other micronutrients (vitamins and minerals), is a major concern for the food industry during food processing operations and storage. Novel nonthermal technologies, namely irradiation, cold plasma, ultrasound, pulsed electric fields (PEF), and high-pressure processing have potential for future food processing and may serve as better alternatives for the retention of nutrients and bioactive compounds, while extending the shelf-life and maintaining the sensory properties of minimally processed potatoes [4].

High-pressure processing (HPP) and ultrasound (US) are emerging non-thermal technologies for food preservation. HPP involves the application of elevated hydrostatic pressure ranging from 100 to 1000 MPa at mild temperatures (<40 °C) for a short time (a few seconds to 20 min) to packaged foods in order to inactivate food-borne microorganisms and enzymes to achieve pasteurization [5,6]. US refers to sonic waves exceeding the human audible frequency range (~20 kHz) that have been developed to minimize processing while ensuring the quality and safety of foods [7]. Currently, HPP and US have been successfully employed to minimally processed foods: HPP altered the structure of cocoyam, Peruvian carrot, and sweet potato leading to higher drying rates in these tubers, while colour parameters remained unaffected in the first two tubers [8,9]. Similarly, no obvious colour changes and decreased respiratory activity were detected in HPP-treated avocado slices [10], while lower enzyme activities associated with browning and anaerobic metabolism were reported in minimally processed peaches after HPP treatment [10]. Amaral et al. (2015) [11,12] reported significant lower polyphenol oxidase activity in US-treated fresh-cut potatoes, while firmness and colour attributes were retained. Another study showed that US had negligible impact on the physicochemical and sensory characteristics of modified atmospheric packaged fresh-cut cucumber during storage [13].

Previous studies from our group have demonstrated that HPP at 600 MPa had minimal effects on the proximate composition, antioxidants, and bioactive compounds of Irish potato cultivars, while it showed promising results regarding polyphenol oxidase inactivation [14,15]. This study is to investigate further the effect of HPP on its own and in combination with US (HPP/US) on the colour and microbial growth of minimally processed potatoes of the two most commonly grown potato varieties (Maris Piper (white cultivar) and Rooster (coloured cultivar)) in Ireland. The total phenolic content (TPC) and antioxidant activity (AOA), as well as the levels of individual phytochemicals, of minimally processed potatoes were also assessed after the HPP and HPP/US treatments.

## 2. Results and Discussion

### 2.1. Effect of HHP and US in Colour of Minimally Processed Potatoes

Colour changes were evaluated by the L*, a*, b*, and ΔE parameters, while colour differences were compared to the untreated samples (controls) on the first day of storage. Results are shown in Table 1.

HPP and HPP followed by US (HPP/US) significantly (*p* < 0.05) promoted the decrease of L* (lightness) value in both potato varieties during storage. The b* (yellowness) values were also reduced (*p* < 0.05) during storage in potatoes of both cultivars after HPP and HPP/US treatment compared to untreated controls. No significant (*p* < 0.05) changes were observed in the values of the a* (redness) parameter in HPP- and HPP/US-treated potato samples of both varieties at day 0 compared to the controls; however, there was an increase (*p* < 0.05) for the rest of the storage period. Similarly, there was an increasing trend in the values of the total colour differences (ΔE), in HPP- and HPP/US-treated samples of both cultivars during storage compared to the controls.

Studies on avocado slices have shown that HPP at 300, 400 and 500 MPa of pressure significantly (*p* < 0.05) decreased lightness (L*) with respect to the untreated controls, similar to this study, whereas at 200 MPa no significant change in L* values was noted [10]. On the contrary, HPP-treated pumpkin slices had higher L* values, although a* and b* parameters were lower in the treated samples than the untreated samples [16]. However, similar to our findings, the total colour difference (ΔE) in HPP-treated pumpkin increased during storage. Even though HPP has a limited impact on compounds such as chlorophyll, carotenoids, and anthocyanins, which are responsible for the colour of fruits and vegetables, these chromophoric compounds can change during storage. The observed alterations may be caused by the incomplete inhibition of enzymes and microorganisms, which can lead to undesired chemical reactions (both enzymatic and non-enzymatic) in the food matrix [17]. Previous work from our group [14] has shown that HPP resulted in a significant (*p* < 0.05) decrease in the polyphenol oxidase (PPO) activity of potatoes; however, the PPO enzyme was not completely inactivated, which could be the cause of the gradual browning of the minimally processed potatoes over time in this study.

Amaral et al. (2015) [11] reported that US had a significant (*p* < 0.05) impact on the lightness (L*) of fresh-cut potatoes after the US treatment and during storage, which is in accordance with our findings. Similar observations were made by the same authors in another study where the combined treatment of US and citric acid (20 gL^−1^) on potato strips resulted in a decrease in L^*^ values of treated potatoes during storage [12]. According to Calder et al. (2011) [18], the decrease in L^*^ value is an indication of browning. An increase in colour parameters (L*, a*, b*) upon ultrasonication was reported in carrot juice [19], apple juice [20], and grapefruit juice [21], while no significant changes in the same parameters were found after a combined treatment of US with high-pressure carbon dioxide in fresh-cut carrots [22]. The influence of US on the stability of the colour parameters is dependent on the particle size, intercellular material release, and pigment stability of the treated product, while possible degradation could also arise due to the cell disruption causing the release of more pigments [23].

### 2.2. Effect of HPP and HPP/US on Microbial Shelf-Life

**Figure 1:**
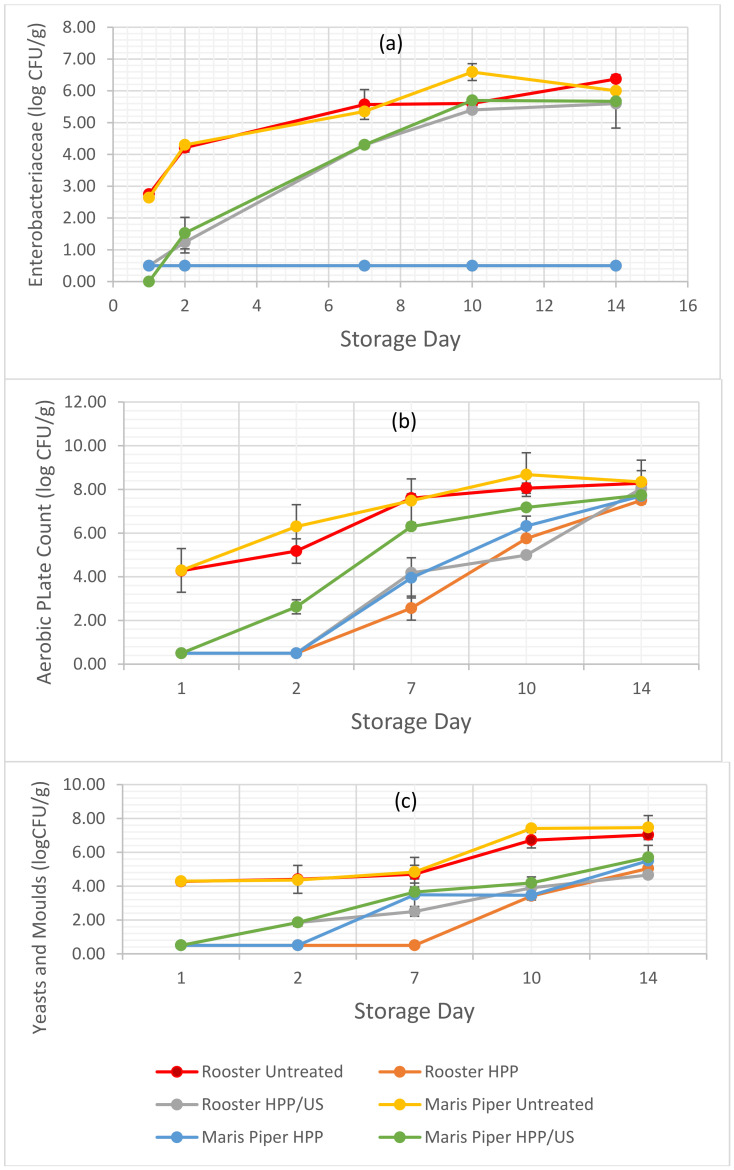
Effect of HPP and HPP/US on *Enterobacteriaceae* (**a**), aerobic plate count (**b**) yeasts and moulds (**c**) in minimally processed potatoes of the Maris Piper and Rooster cultivars during storage.

The impact of HPP and HPP/US treatment on microbial counts can be seen in Figure 1a–c below:

*Enterobacteriaceae* were not detectable in the HPP-treated samples of both potato cultivars. Although HPP followed by US slowed down the growth of these microorganisms, it did not have a significant (*p* > 0.05) effect on them by the end of the storage period when compared to the untreated samples. Similarly, there was a significant (*p* < 0.05) retardation of the aerobic plate count (APC) in HPP- and HPP/US-treated samples of both cultivars until the 10th day of storage. In fact, HPP/US-treated Rooster samples had significantly lower APC than the controls and the HPP-treated samples until the 10th day of storage, suggesting that the combination of HPP and US was more efficient on this occasion. However, by the end of the storage period there was no significant (*p* > 0.05) difference between the treated and untreated potatoes of both varieties. HPP and HPP/US treatments had a significant (*p* < 0.05) effect on yeasts and moulds; treated samples of both cultivars exhibited a delay in growth during the first days of storage, while they had a lower count of yeasts and moulds compared to those untreated by the end of the storage time. *Salmonella* was absent in all studied samples throughout the storage period.

Similar to the findings of this study, HPP treatment had resulted in a significant decrease in the total aerobic bacterial count and the yeast and mould counts in pumpkin slices during storage [16]. The same authors also noted that the bactericidal effect was enhanced significantly with the rise in applied pressure and the duration of the treatment. Wang et al. (2012) [24] reported a significant reduction in the total aerobic bacteria, yeasts, and moulds in sweet potato nectar after the application of HPP at 400, 500, and 600 MPa compared to the control. These authors observed no significant difference amongst the results obtained from the different HPP treatments. The inhibition of aerobic bacteria by HPP is considered to be the result of a combination of morphological alterations in microbial cells, such as membrane disruption and loss of its function, compression of gas vacuoles, cell expansion, formation of pores in the cell wall, and the destruction of ribosomes [25]. Numerous factors affect the inactivation of microorganisms by HPP including the type of microorganism, composition of suspension media or food, applied pressure, duration of treatment, pH, water activity (a_w_), temperature, and the redox potential of the pressure menstruum [26].

Amaral et al. (2017) [12] noted that the combined treatment of US and citric acid (20 gL^−1^) on potato strips resulted in the reduction of total coliforms, *Enterobacteriaceae*, and aerobic bacteria, while there was also a slight decrease of yeasts and moulds after treatment, similar to the findings of this study. The authors attributed the inactivation of microorganisms to a combination of physical and chemical mechanisms, due to cavitation, which leads to the formation of free radicals and hydrogen peroxide [12]. Zudaire et al. (2019) [27] reported that, although US treatment for 10 min did not significantly decrease the total aerobic count in calçots, when applied for 45 min, it led to a significant reduction of the microbial load in the treated samples when compared to those untreated. The impact of US on microorganisms is dependent on (i) external factors, namely, US frequency, power, and amplitude, (ii) intrinsic factors, such as the food matrix, as well as the structure and composition of it, and (iii) the type and characteristics of the microorganism [23].

### 2.3. Effect of HPP and HPP/US on Total Phenolic Content

**Figure 2:**
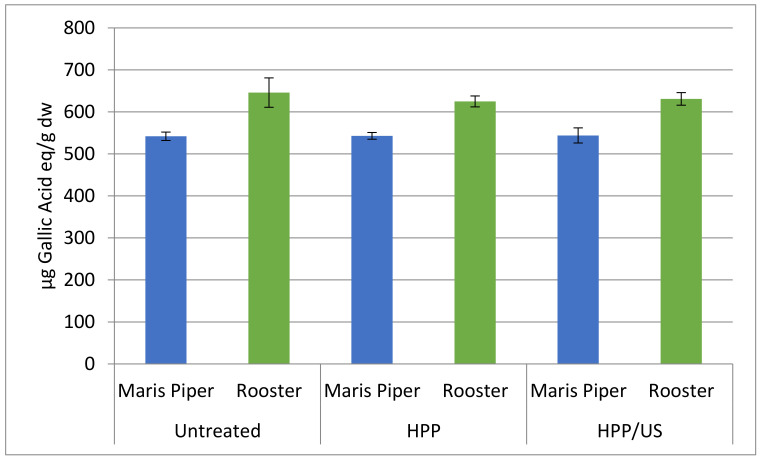
The effect of HPP and HPP/US treatment on total phenolic content expressed as µg gallic acid equilavent per gram dry weight of minimally processed potatoes of the Maris Piper and Rooster varieties.

Figure 2 presents the impact of HPP and HPP/US on total phenolic content (TPC) of minimally processed potatoes of the Rooster and Maris Piper cultivars. The TPC in the untreated Maris Piper potatoes was 542 ± 10 μg GAE/g dry weight (dw), while in the Rooster cultivar, it was 646 ± 35 μg GAE/g dw, which is consistent with the literature [28]. No significant (*p* > 0.05) changes were observed in the HPP- and HPP/US-treated samples compared to the untreated samples. These results were different than in our previous study [14], where a significant (*p* < 0.05) increase of TPC was observed in whole potatoes after the HPP at 600 MPa. However, it should be taken into consideration that the levels of phenolic compounds and their stability, and therefore, the influence of a processing technology on them are affected by various parameters, such as genetic (cultivar), environmental, and agronomic factors, as well as the stage of ripeness and postharvest handling and storage [3,29]. HPP has also resulted in insignificant changes in mango nectars [30], litchi juice, [31] and acai juice [32] when compared to untreated controls. On the other hand, TPC in pumpkin slices was increased significantly after HPP at 450 MPa for 15 min and 550 MPa for 10 min [16], while higher TPC was observed in HPP-treated pomegranate juice as compared to untreated juice [33]. The TPC increment that was detected in both studies was attributed to enhanced cell permeability caused by the disruption of the cell walls as well of the cell membrane hydrophobic bonds, resulting in mass transfer and the release of matrix-bound phenolic compounds.

Similar findings to this study were also reported by Wang et al. (2015) [34], where US treatment at 20 kHz and at three different power densities (66.64, 106.19, and 145.74 W/L) led to insignificant changes in the TPC of cherry tomatoes. On the other hand, Ferrentino et al. (2015) [35] found lower TPC levels in fresh-cut coconut after the application of a combination of high-power US (30 kHz, 40 W, 30 min) and high-pressure carbon dioxide (12 MPa, 35 °C). However, these findings could not be attributed directly to the ultrasonic effect because the impact of each treatment separately was not examined in their study. On the other hand, lower levels of TPC were found in red raspberry puree after sonication for 30 min at 490 kHz [36], while a decrease in TPC was detected in model systems using US at 358 and 1062 kHz [37]. The latter findings were correlated with an observed decrease in hydroxyl radicals and an increased concentration of hydroxylated products. Conversely, a significant increase was observed in the TPC of fresh-cut pineapples sonicated at a constant frequency of 37 kHz, but at different power inputs (27 and 29 W), for 10 and 15 min with respect to the controls; this correlated with an increase in the activity of phenylalanine ammonia lyase [38].

### 2.4. Effect of HPP and HPP/US on Antioxidant Activity

**Figure 3:**
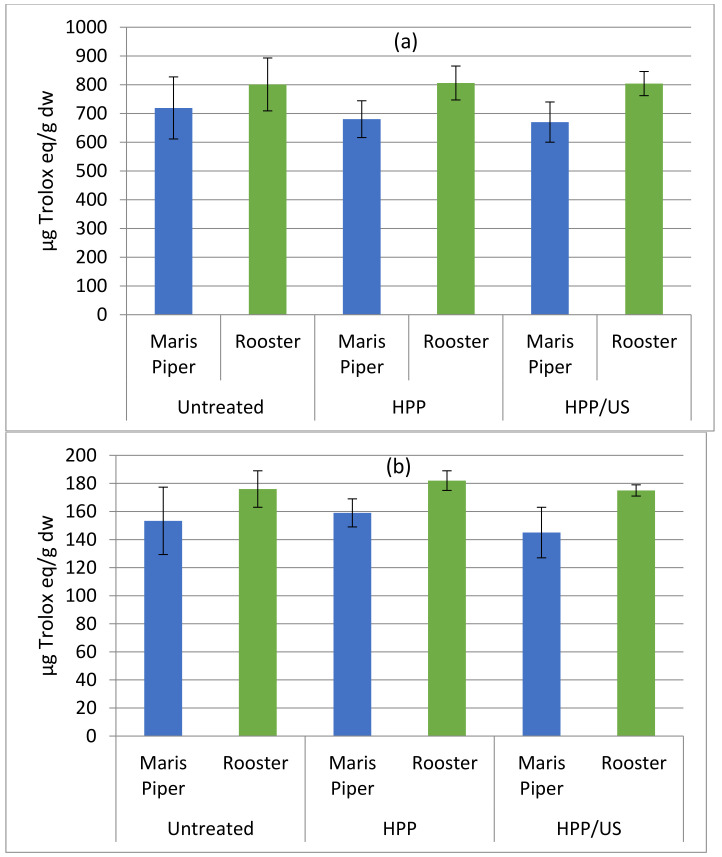
FRAP (**a**) and DPPH radical scavenging capacity (**b**) expressed as μg Trolox equivalent (TE)/g dry weight in untreated, HPP- and HPP/US-treated minimally processed potatoes of the Maris Piper and Rooster cultivars.

The antioxidant activities of untreated, HPP- and HPP/US-treated minimally processed potatoes, determined using FRAP and DPPH radical scavenging assays for Maris Piper and Rooster, are shown in Figure 3a,b, respectively. The antioxidant activity (AOA) of minimally processed potatoes, estimated by both the assays, varied depending on the cultivar and the type of the treatment, but changes were statistically insignificant after HPP or HPP/US treatment. These results are consistent with a previously published study on the impact of HPP at 600 MPa on the AOA of Irish potato cultivars [15]. Insignificant (*p* > 0.05) changes had been observed in the AOA of aronia berry puree after HPP at 400 and 600 MPa [39], in mango nectars after HPP at 600 MPa [30], and in sweet potato nectar [24]. The authors of the latter study also associated their findings with insignificant changes in the total phenolic and total anthocyanin contents. On the other hand, a slight but significant (*p* < 0.05) increase in AOA after HPP treatment has been detected in smoothies [40] and in pumpkin [16]. According to Briones-Labarca et al. (2011) [41], the increase in the antioxidant activity of a plant tissue extract upon HPP treatment can be attributed to the disruption of plant cell walls resulting in the release of antioxidant compounds into the extracellular environment. Nevertheless, the authors reported lower AOA in Granny Smith apples treated at 500 MPa for 2–10 min compared to untreated apple samples [41]. Interestingly, the HPP-treated apple samples prior to in vitro digestion had shown higher AOA as opposed to those untreated. The authors postulated that the amount of antioxidants released by the plant matrix into the human intestine, and consequently, the AOA of these compounds, may be higher than the data obtained from chemical extracts [41]. However, a significant (*p* < 0.05) reduction in AOA was reported in aloe vera gel after HPP at 150, 250, 350, 450, and 550 MPa for 5 min [42]. These contradictory findings suggest that the impact of HPP on AOA is very much reliant on the matrix studied.

As observed in the current study, no significant (*p* > 0.05) differences in the AOA of cherry tomatoes were found between those sonicated at 60.45 W/L and untreated samples [34]. Insignificant changes in the AOA as assayed by the DPPH radical scavenging method were also observed in the US-treated purple cactus pear fruits [43] and red raspberry puree [36] as compared to controls. On the other hand, a higher reducing power (Fe^3+^–Fe^2+^ transformation) was detected in chokanan mango juice upon sonication (40 kHz, 130 W), which was attributed to the removal of occluded oxygen [44]. Similarly, a significant increment in total antioxidant capacity of fresh-cut pineapple, as examined by the FRAP assay, was reported in sonicated samples at 37 kHz and 25 or 29 W for 10 to 15 min compared to the controls [38]. Furthermore the AOA correlated with the observed increase in the TPC of US-treated pineapples, indicating that the polyphenols are the main antioxidants affecting the AOA of pineapples [38].

The aforementioned results indicate a nondestructive influence of HPP and US on the antioxidant compounds of foods.

### 2.5. Effect of HPP and HPP/US on Phytochemicals

The impact of HPP and HPP/US on the phytochemical content of minimally processed potatoes of the Maris Piper and Rooster varieties is presented in Table 2. Chlorogenic acid was the most abundant polyphenol in the untreated samples, and it ranged from approximately 81 to 97 μg/g dw, which is in good agreement with existing literature [45]. HPP and HPP/US treatments significantly (*p* < 0.05) decreased chlorogenic acid in both studied cultivars, which is consistent with previous findings [14]. A simultaneous increase (*p* < 0.05) of quinic acid was also observed, which has been previously reported [14], while the concentration of caffeic acid was decreased in the treated samples. Ferulic acid was significantly (*p* < 0.05) decreased in the HPP/US-treated Maris Piper samples, while the rest of the treated samples had insignificant changes in the levels of ferulic acid compared to the control samples. A similar trend where the levels of chlorogenic acid, caffeic acid, and ferulic acid, examined in tomato purée samples, were significantly reduced in HPP (450, 550, and 650 MPa, 5 and 15 min)-treated samples when compared to the controls [46]. The authors concluded that the applied pressure as well as the treatment duration were responsible for the reduction. Wang et al. (2020) [47] recently investigated the degradation behaviour of three polyphenols, namely, caffeic acid, rutin and cyanidin-3-glucoside, upon mechanical and sonochemical effects caused by sonication at 20 kHz and 120 W. The authors concluded that the degradation of the polyphenols was mainly induced by the hydroxyl radicals produced by the US, which could explain the observed decrease in the levels of the polyphenols in the HPP/US-treated samples of this study.

Steroidal alkaloids (glycoalkaloids) are nitrogenous secondary metabolites that are present in potatoes and other members of the Solanaceae family. The main glycoalkaloids in potatoes are α-chaconine and α-solanine, constituting about 95% of the total glycoalkaloid content. The concentrations of α-chaconine and α-solanine ranged from 1.06–1.52 µg/g and 1.10–1.73 µg/g, respectively, in untreated samples of both cultivars, which is in accordance with the literature [31]. Both HPP and HPP/US treatments significantly decreased (*p* < 0.05) the glycoalkaloid content of the studied varieties. HPP led to a 72% decrease of α-chaconine and α-solanine in the Maris Piper cultivar, while HPP/US decreased these glycoalkaloids by approximately 97%, as compared to the untreated samples. A 55% reduction of α-chaconine was observed in Rooster potatoes upon HPP, while α-solanine was decreased by 66% in the HPP-treated Rooster samples with respect to untreated controls. HPP/US resulted in a 95% decrease of the glycoalkaloid content of Rooster potatoes as compared to the controls. Numerous factors affect the formation of glycoalkaloids in potatoes: environmental and growing conditions, maturity during harvesting time, temperature during growth, and extent of sprouting, as well as any mechanical damage such as bruising, cutting, wounding, and slicing while handling. Postharvest storage conditions, especially the wavelength, duration, and intensity of light during storage can also play a crucial role on the glycoalkaloid content of potatoes, while other environmental conditions during packaging, transportation, and marketing can have an effect on it as well [48,49]. A previous study [14] found no statistically significant changes to the glycoalkaloid content of whole potatoes after HPP treatment. Since the glycoalkaloids are present on the skin (usually no deeper than 3 mm) [50,51], minimal processing can result in their decrease and the HPP/US could also trigger degradation to aglycone alkaloids, which can explain the contradiction between the findings of the current study and those from previous work.

## 3. Materials and Methods

### 3.1. Fresh Potatoes

Potatoes (*Solanum tuberosum* L.) of the Maris Piper and Rooster cultivar were purchased from a local market in Dublin, Ireland.

### 3.2. Chemicals

Trolox, methanol, hydrochloric acid (HCl), anhydrous sodium acetate, acetic acid, 2,3,5-triphenyltetrazolium chloride (TPTZ), ferrous (Iron II) chloride hexahydrate, 2,2-diphenyl-1-picrylhydrazyl (DPPH), gallic acid, sodium carbonate, and Folin–Ciocalteau reagent (FCR) were purchased from Merck, Wicklow, Ireland. The microbiological media were purchased from Oxoid, Basingstoke, UK.

### 3.3. HPP/Ultrasound Treatment

Potato tubers of the two cultivars, free of defects, were washed, hand-peeled, packaged in polyethylene/polyamide pouches and then vacuum sealed. HPP treatment was performed at 600 MPa (6000 bar) for 3 min at 10.6 °C (maximum temperature reached) as described in previous studies [14,15]. A commercial-scale high-pressure process (Hiperbaric 55HT, Miami, FL, USA) was used, located at HPP Tolling (St. Margaret’s, Co. Dublin, Ireland).

After HPP treatment, the potatoes (~150 g) were cut into cubes with dimensions of 2 × 2 × 2 cm and subjected to sonication (Extractor 200, Idco, Marseille, France) at 20 kHz and 200 W for 10 min. The US conditions as well as the treatment duration were chosen following preliminary experimental studies (data not shown). The starting temperature of the US treatment was 18 °C, while the maximum temperature reached after the treatment was 28 ± 2 °C.

### 3.4. Colourimetry

Colour changes in untreated, HPP- and HPP/US-treated minimally processed potato samples of Rooster and Maris Piper cultivars were monitored using a colourimeter (HunterLab UltraScan Pro, Hunter Associates Laboratory, Inc., Reston, VA, USA). The colour parameters, lightness (L*), redness (a*), and yellowness (b*), were measured for the days 0, 1, 7, 10, and 14 from the initial treatment of samples. Measurements were taken from two separate sides of the potato cubes, at three different points (*n* = 6).

The colour differences were calculated as the Euclidean distance between two points in the three-dimensional space, using the following formula:

$$\Delta E=\sqrt{{\left({\Delta L}^{*}\right)}^{2}+{\left({\Delta a}^{*}\right)}^{2}+{\left({\Delta b}^{*}\right)}^{2}}$$

where ΔL*, Δa*, and Δb* are differences between untreated and treated potato samples.

### 3.5. Microbial Shelf-Life Assessment

The microbiological safety of minimally processed potato samples was investigated during storage (Day 1, 2, 7, 10, and 14). In brief, approximately 10 g of potato was weighed into a stomacher bag and a primary 10-fold dilution was performed by the addition of 90 mL of sterile maximum recovery diluent (MRD), stomached for 1 min, and homogenates were 10-fold serially diluted using the MRD solution.

The following cultural methods were employed:

- *Enterobacteriaceae* based on ISO 21528:2017 (International Standards Organisation. ISO 21528-2:2017 Microbiology of the food chain—Horizontal method for the detection and enumeration of *Enterobacteriaceae*—Part 2: Colony-count technique): Sample dilutions were mixed with violet red bile glucose (VRBG) agar at 50 °C and after solidification they were covered with another layer of VRBG. Plates were incubated at 37 °C for 24 h.
- Yeasts and moulds count based on ISO 21527-1 (International Standards Organisation. ISO 21527-1:2008 Microbiology of food and animal feeding stuffs—Horizontal method for the enumeration of yeasts and moulds—Part 1: Colony-count technique in products with water activity greater than 0.95): pour plate method using oxytetracycline glucose yeast extract agar base with oxytetracycline supplement (OGYE). Plates were incubated for five days at 25 °C, with initial counts taken at three and four days.
- Aerobic plate count based on ISO 4833 (International Standards Organisation. ISO 4833-1:2013 Microbiology of the food chain—Horizontal method for the enumeration of microorganisms—Part 1: colony count at 30 °C by the pour plate technique): spread plate method on plate count agar. Plates are incubated for three days at 30 °C.
- Absence of *Salmonella* based on ISO 6579 (International Standards Organisation. ISO 6579-1:2017 Microbiology of the food chain—Horizontal method for the detection, enumeration and serotyping of salmonella—Part 1: Detection of *Salmonella* spp.): Samples were pre-enriched overnight in buffered peptone water, before 100 μL of each sample was pipetted onto modified semi-solid Rappaport-Vassiliadis (MSRV) agar plates and incubated at 42 °C for 24 h. If the MSRV plates were negative, they were incubated for a further 24 h. Presumptive *Salmonella* growth would be streaked onto xylose lysine deoxycholate (XLD) and brilliant green (BG) agar and incubated at 37 °C for 24 h with subsequent serological confirmation.

In all cases results were expressed as log_10_ CFU/g.

### 3.6. Bioactive Compounds Extraction

Phytochemicals were extracted according to a previously published study [14]. Briefly, freeze-dried potato samples (~0.2 g) were mixed with methanol (1 mL, 80% *v*/*v*) containing formic acid (1% *v*/*v*) and kept overnight at 4 °C. Afterwards, the mixture was sonicated at 30 °C for 30 min, and centrifuged at 10,000× g for 30 min. The residue was re-extracted with methanol (0.8 mL, 80% *v*/*v*) containing formic acid (1% *v*/*v*), followed by centrifugation. The supernatants were combined and filtered through a 0.45 µm syringe filter.

### 3.7. Total Phenolic Content

A modified version of the Folin–Ciocalteu reagent (FCR) method [52], in order to be performed in a microplate reader (FLUOstar Omega Microplate Reader, BMG Labtech GmbH, Offenburg, Germany), was used for the determination of the total phenolic content (TPC) of potatoes, as previously mentioned in [14]. In brief, potato extract (100 µL) was mixed with FCR (100 µL), sodium carbonate (100 µL; 20% *w*/*v*), and methanol (100 µL). The mixture was held in the dark for 20 min, followed by centrifugation at 13,000 rpm for 3 min. The absorbance was measured at 735 nm in the microplate reader using gallic acid as a standard and methanol as a blank. The results were expressed as µg of gallic acid equivalent per g of dry weight of the extract (µg GAE/g dw).

### 3.8. Determination of Antioxidant Activity (AOA)

#### 3.8.1. Ferric Reducing Antioxidant Power (FRAP)

The FRAP assay was assayed using a modified method of Benzie and Strain (1996) [53] by Stratil et al. (2006) [54] and Ou et al. (2002) [55]; Potato extract (20 μL) was mixed with a FRAP solution (180 μL) that was prepared by mixing acetate buffer (100 mL; 0.3 M, pH 3.6) with ferrous chloride hexahydrate (10 mL; 0.01 M) and TPTZ (10 mL; 0.01M in 0.04 M HCl) and incubating it at 37 °C for 40 min. The absorbance was monitored at 593 nm in the microplate reader using Trolox as a standard and methanol as a blank. The results were expressed as µg of Trolox equivalent per g of dry weight of the extract (µg TE/g dw).

#### 3.8.2. DPPH Radical Scavenging Capacity

The DPPH radical scavenging capacity was calculated according to Goupy et al. (1999) [56]. DPPH (11.9 mg) was mixed with methanol (50 mL). A 1:5 dilution of the DPPH stock solution was prepared. Potato extract (100 μL) was blended with the diluted DPPH solution (100 μL) and kept in the dark for 30 min before reading the absorbance at 515 nm in the plate reader. Trolox was used as a standard and methanol as a blank. The results were expressed as µg of Trolox equivalent per g of dry weight of the extract (µg TE/g dw).

### 3.9. Liquid Chromatography-Mass Spectrometry Analysis

Phytochemicals previously identified by HPLC-QToF mass spectrometry [14,57] were quantified by ultra-high performance liquid chromatography coupled to a tandem quadrupole mass spectrometer (UPLC-TQD, Waters Corp., Milford, MA, USA). A Waters Acquity UPLC HSS T3 column (100 × 2.1 mm, 1.8 µm) was employed in order to separate the natural compounds in the potato extract. Water containing formic acid (0.1%) and formic acid (0.1%) in acetonitrile were used as solvents at a 0.5 mL/min flow rate [58,59]. Detection and quantification of the phenolic compounds in the UPLC-TQD were performed in multiple reaction monitoring (MRM) mode by analysing at least two transitions per compound. The cone voltages and collision energies were optimised for MRM transition ions while using IntelliStart^T^ software (Masslynx 4.1, Waters Corp., Milford, MA, USA). Analyses were carried out in quadruplicate extracts and target compounds were quantified using standard calibration curves of concentrations that ranged from 10 ng/mL–25 µg/mL. The results were expressed as µg compound per g of extract in dry weight (µg/g dw).

### 3.10. Statistical Analysis

Experiments were carried out in duplicate while all analyses were replicated three times (*n* = 6). Results are expressed as means ± standard deviation (SD). One-way ANOVA and Tukey’s post hoc test were performed for the statistical analysis of the data. All of the statistical analyses were performed by SPSS Statistics 26 (IBM-Armonk, New York, NY, USA). The values were considered to be significantly different when *p* < 0.05.

## 4. Conclusions

High-pressure processing (HPP) alone and in combination with ultrasound (HPP/US) had a significant impact on the colour parameters (a*, b*, L*, ΔE) of minimally processed potatoes compared to the controls that were not subjected to physical processing. HPP completely inhibited the growth of *Enterobacteriaceae* in both the potato cultivars, while most of the HPP- or HPP/US-treated samples exhibited a delay in microbial growth as compared to those untreated, thus meriting the commercial use of HPP as a decontamination process. Inclusion of US can further eliminate the aerobic bacteria and thereby, assure the microbial safety of the product. Although levels of individual (poly)phenols were altered after HPP and HPP/US, the total phenolic content and antioxidant activity remained unaltered and thereby, retained the health-promoting compounds. A significant decrease in the levels of the cytotoxic glycoalkaloids α-chaconine and α-solanine in HPP- and HPP/US-treated potatoes would further benefit the consumers. In general, these findings demonstrate that HPP and US could prolong the shelf-life of minimally processed potatoes with a minimal effect on their antioxidant activity and phenolic content. Further research is needed in order to deepen the understanding of the microbial inactivation mechanism as well as to diminish the impact of these technologies on the colour and shelf-life of minimally processed potatoes.

## Figures and Tables

**Table 1:** Values of colour parameters in untreated (controls), HPP- and HPP/US-treated minimally processed potatoes of the Maris Piper and Rooster cultivars.

Sample	Treatment	Colour	Day of Storage				
			0	1	7	10	14
Maris Piper	untreated	L*	64.16 ± 2.16 ^a^	62.24 ± 4.28 ^a^	59.47 ± 1.96 ^b^	62.52 ± 3.81 ^a^	62.41 ± 4.38 ^a^
	HPP		66.56 ± 2.54 ^a^	61.00 ± 4.95 ^a^	53.35 ± 2.05 ^b^	59.95 ± 2.01 ^b^	56.15 ± 3.64 ^b^
	HPP/US		61.17 ± 3.47 ^a^	53.77 ± 5.39 ^b^	49.72 ± 1.54 ^b^	49.19 ± 3.02 ^b^	49.03 ± 4.19 ^b^
	untreated	a*	−0.13 ± 0.36 ^a^	1.01 ± 0.10 ^b^	2.32 ± 0.67 ^a b^	2.60 ± 0.84 ^a b^	3.00 ± 1.05 ^a b^
	HPP		−0.36 ± 0.66 ^a^	2.01 ± 1.18 ^b^	2.20 ± 0.46 ^b^	3.34 ± 0.51 ^b^	4.22 ± 0.56 ^a b^
	HPP/US		−0.83 ± 0.54 ^a^	2.25 ± 1.27 ^b^	3.03 ± 0.18 ^a b^	2.72 ± 0.26 ^a b^	2.59 ± 0.17 ^a b^
	untreated	b*	14.49 ± 1.32 ^a^	13.79 ± 1.51 ^a^	15.23 ± 0.89 ^a^	16.05 ± 2.53 ^a^	15.58 ± 1.57 ^a^
	HPP		12.59 ± 2.79 ^a^	12.20 ± 3.20 ^a^	10.47 ± 0.24 ^b^	12.11 ± 1.26 ^a^	12.86 ± 1.50 ^a^
	HPP/US		12.14 ± 1.65 ^a^	10.48 ± 0.97 ^b^	8.09 ± 0.75 ^c^	7.49 ± 0.99 ^c^	7.42 ± 0.90 ^c^
	untreated	ΔE		3.14 ± 1.01 ^a^	5.44 ± 0.60 ^b^	4.10 ± 0.69 ^a^	4.39 ± 0.83 ^a^
	HPP		3.38 ± 0.55 ^a^	6.89 ± 1.95 ^b^	13.88 ± 1.72 ^c^	7.11 ± 1.51 ^b^	10.10 ± 1.48 ^d^
	HPP/US		5.79 ± 0.61 ^b^	14.09 ± 1.80 ^c^	22.07 ± 1.16 ^e^	17.55 ± 3.65 ^f^	18.96 ± 2.43 ^f^
Rooster	untreated	L*	63.98 ± 3.11 ^a^	61.31 ± 3.56 ^a^	59.91 ± 2.51 ^a^	60.07 ± 3.09 ^a^	59.13 ± 3.12 ^a^
	HPP		61.61 ± 2.25 ^a^	58.26 ± 2.79 ^a^	47.89 ± 3.11 ^b^	45.73 ± 2.49 ^b^	47.13 ± 2.32 ^b^
	HPP/US		63.17 ± 2.92 ^a^	61.05 ± 4.74 ^a^	48.15 ± 3.86 ^b^	50.87 ± 1.84 ^b^	48.11 ± 1.06 ^b^
	untreated	a*	−0.78 ± 0.47 ^a^	0.26 ± 0.48 ^a^	2.44 ± 0.65 ^b^	2.59 ± 0.65 ^b^	2.97 ± 0.58 ^b^
	HPP		−0.40 ± 1.34 ^a^	3.62 ± 1.59 ^b^	2.20 ± 0.65 ^b^	2.20 ±0.61 ^b^	3.69 ± 1.57 ^b^
	HPP/US		−0.86 ± 0.99 ^a^	1.17 ± 1.04 ^a^	2.06 ± 0.49 ^b^	2.60 ± 0.18 ^b^	3.68 ± 1.12 ^b^
	untreated	b*	23.33 ± 3.07 ^a^	21.38 ± 3.12 ^a^	20.13 ± 4.43 ^a^	19.47 ± 1.26 ^a^	18.70 ± 3.20 ^a^
	HPP		18.19 ± 2.01 ^a^	17.35 ± 3.24 ^a^	12.18 ± 1.60 ^b^	12.47 ± 2.39 ^b^	11.24 ± 1.71 ^b^
	HPP/US		15.32 ± 1.96 ^b^	14.48 ± 2.54 ^b^	8.86 ± 2.39 ^c^	11.68 ± 0.97 ^c^	10.94 ± 0.93 ^c^
	untreated	ΔE		3.58 ± 0.97 ^a^	8.19 ± 1.16 ^b^	6.95 ± 0.78 ^b^	8.06 ± 0.84 ^b^
	HPP		5.79 ± 1.56 ^b^	8.43 ± 1.30 ^b^	19.98 ± 1.23 ^c^	18.77 ± 1.32 ^c^	21.23 ± 1.61 ^c^
	HPP/US		8.07 ± 1.61 ^b^	9.71 ± 2.36 ^b^	21.71 ± 1.40 ^c^	19.17 ± 1.05 ^c^	18.03 ± 1.57 ^c^

All data are the means ± SD (*n* = 6). Values with different letters within a column and same variety are significantly different (*p* < 0.05).

**Table 2:** Phytochemical content (µg/g dry weight) in untreated, HPP- and HPP/US-treated minimally processed potatoes.

	Ferulic Acid	Chlorogenic Acid	Quinic Acid	Caffeic Acid	α-Chaconine	α-Solanine
Maris Piper Untreated	50.41 ± 4.44 ^a^	80.60 ± 6.4 ^b^	24.12 ± 1.5 ^a^	3.45 ± 0.11 ^a^	1.52 ± 0.27 ^a^	1.73 ± 0.62 ^a^
Maris Piper HPP	25.18 ± 2.52 ^b^	28.38 ± 6.7 ^c^	28.14 ± 1.66 ^b^	2.70 ± 0.27 ^b^	0.42 ± 0.09 ^b^	0.48 ± 0.05 ^b^
Maris Piper HPP/US	29.21 ± 1.57 ^b^	3.31 ± 0.21 ^e^	35.07 ± 1.55 ^c^	0.51 ± 0.06 ^d^	0.05 ± 0.02 ^c^	0.06 ± 0.005 ^c^
Rooster Untreated	56.47 ± 4.35 ^a^	97.19 ± 1.7 ^a^	26.51 ± 1.24 ^a^	4.76 ± 1.08 ^a^	1.06 ± 0.38 ^a^	1.10 ± 0.40 ^a^
Rooster HPP	50.77 ± 2.93 ^a^	15.85 ± 0.4 ^d^	28.76 ± 0.12 ^b^	1.21 ± 0.63 ^c^	0.47 ± 0.06 ^b^	0.37 ± 0.02 ^b^
Rooster HPP/US	35.48 ± 1.75 ^c^	1.78 ± 0.21 ^f^	33.25 ± 1.02 ^c^	0.48 ± 0.08 ^d^	0.06 ± 0.01 ^c^	0.06 ± 0.01 ^c^

All data are the means ± SD (*n* = 6). Values with different letters within the same column are significantly different (*p* < 0.05).

## Data Availability

Data available in a publicly accessible repository.

## Sample Availability Statement

Samples of the compounds are not available from the authors 4/29 Ema.
